# Factors Predicting Treatment of World Trade Center-Related Lung Injury: A Longitudinal Cohort Study

**DOI:** 10.3390/ijerph17239056

**Published:** 2020-12-04

**Authors:** Barbara Putman, Lies Lahousse, David G. Goldfarb, Rachel Zeig-Owens, Theresa Schwartz, Ankura Singh, Brandon Vaeth, Charles B. Hall, Elizabeth A. Lancet, Mayris P. Webber, Hillel W. Cohen, David J. Prezant, Michael D. Weiden

**Affiliations:** 1 Department of Bioanalysis, Faculty of Pharmaceutical Sciences, Ghent University, 9000 Ghent, Belgium; Barbara.Putman@Ugent.be (B.P.); Lies.Lahousse@Ugent.be (L.L.); 2 Pulmonary, Critical Care and Sleep Medicine Division, Department of Medicine and Department of Environmental Medicine, New York University School of Medicine, New York, NY 10016, USA; 3 The Bureau of Health Services and the FDNY World Trade Center Health Program, Fire Department of the City of New York, Brooklyn, NY 11201, USA; David.Goldfarb@fdny.nyc.gov (D.G.G.); Rachel.Zeig-Owens@fdny.nyc.gov (R.Z.-O.); Theresa.Schwartz@fdny.nyc.gov (T.S.); Ankura.Singh@fdny.nyc.gov (A.S.); Brandon.Vaeth@fdny.nyc.gov (B.V.); Mayris.Webber@fdny.nyc.gov (M.P.W.); David.Prezant@fdny.nyc.gov (D.J.P.); 4 Pulmonary Medicine Division, Department of Medicine, Montefiore Medical Center and Albert Einstein College of Medicine, Bronx, NY 10467, USA; 5 Division of Epidemiology, Department of Epidemiology and Population Health, Albert Einstein College of Medicine, Bronx, NY 10461, USA; Hillel.Cohen@einsteinmed.org; 6 Division of Biostatistics, Department of Epidemiology and Population Health, Albert Einstein College of Medicine, Bronx, NY 10461, USA; Charles.Hall@einsteinmed.org; 7 The Office of Medical Affairs, Fire Department of the City of New York, Brooklyn, NY 11201, USA; Elizabeth.Lancet@fdny.nyc.gov

**Keywords:** inhalation therapy, pulmonary function tests, lung injury, occupational exposure, epidemiological studies

## Abstract

The factors that predict treatment of lung injury in occupational cohorts are poorly defined. We aimed to identify patient characteristics associated with initiation of treatment with inhaled corticosteroid/long-acting beta-agonist (ICS/LABA) >2 years among World Trade Center (WTC)-exposed firefighters. The study population included 8530 WTC-exposed firefighters. Multivariable logistic regression assessed the association of patient characteristics with ICS/LABA treatment for >2 years over two-year intervals from 11 September 2001–10 September 2017. Cox proportional hazards models measured the association of high probability of ICS/LABA initiation with actual ICS/LABA initiation in subsequent intervals. Between 11 September 2001–1 July 2018, 1629/8530 (19.1%) firefighters initiated ICS/LABA treatment for >2 years. Forced Expiratory Volume in 1 s (FEV_1_), wheeze, and dyspnea were consistently and independently associated with ICS/LABA treatment. High-intensity WTC exposure was associated with ICS/LABA between 11 September 2001–10 September 2003. The 10th percentile of risk for ICS/LABA between 11 September 2005–10 Septmeber 2007 was associated with a 3.32-fold increased hazard of actual ICS/LABA initiation in the subsequent 4 years. In firefighters with WTC exposure, FEV_1_, wheeze, and dyspnea were independently associated with prolonged ICS/LABA treatment. A high risk for treatment was identifiable from routine monitoring exam results years before treatment initiation.

## 1. Introduction

Rescue and recovery workers suffered a massive exposure to dust and products of combustion after the collapse of the World Trade Center (WTC) [1]. This resulted in increased rates of respiratory symptoms, as well as an acute drop of lung function and subsequent high incidence of reactive airways disease and fixed airflow obstruction [2,3,4,5,6,7,8]. Immediately after 11 September 2001 (9/11), the Fire Department of the City of New York (FDNY) instituted a medical monitoring program that performed screening pulmonary function tests and collected respiratory symptom data via computer-based questionnaires [9]. Additionally, FDNY began a treatment program at no cost to the participants. Longitudinal data from these monitoring and treatment programs have identified WTC-related diseases and treatment responses. We previously found that initiation of treatment with inhaled steroids combined with long-acting beta agonists (ICS/LABA) sooner after WTC exposure was more likely to result in improvement of dyspnea in the FDNY cohort than initiation of treatment years after WTC exposure [10]. 

ICS/LABA is the standard of care for asthma and Chronic Obstructive Pulmonary Disease (COPD) with exacerbations [11,12]. Current guidelines on obstructive airway diseases recommend that physicians evaluate lung function, respiratory symptoms, and frequency of exacerbations to inform treatment strategies [13,14]. In the era of telemedicine, web-based symptom monitoring, wearable devices, and data transmission technology are becoming more widely used [15,16]. Predictive models using these data will help detect individuals with undiagnosed disease who may benefit from early interventions such as subspecialty testing and treatment.

It remains unclear which factors identify at-risk individuals prior to the development of abnormal lung function on screening spirometry exams. A subgroup of individuals has experienced an accelerated decline of lung function, but the majority still have spirometry measurements within normal limits [7]. The objective of this study was therefore to identify patient characteristics collected on routine medical monitoring that would predict WTC-related lung injury defined by initiation of ICS/LABA treatment for more than 2 years.

## 2. Materials and Methods 

### 2.1. Source Population and Data Sources

**Figure 1:**
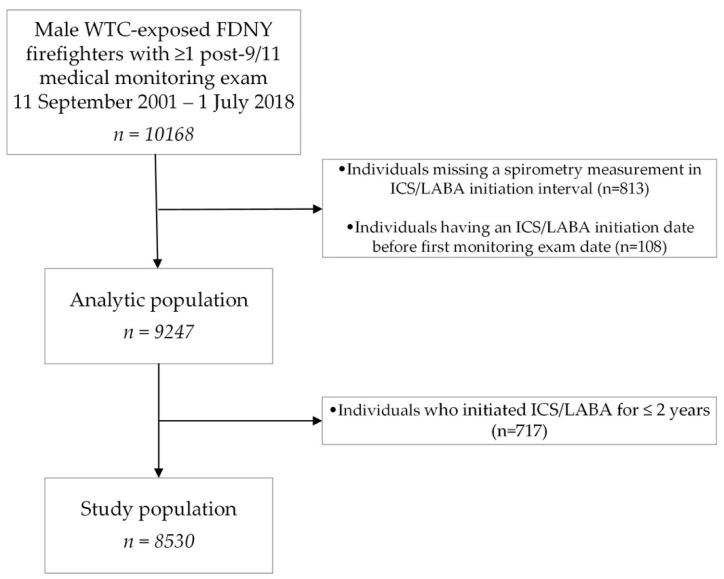
Flowchart of the study population. The source population included 10168 World Trade Center (WTC)-exposed male firefighters who were actively employed by FDNY on 11 September 2001, consented to research, and had at least one routine medical monitoring exam between 11 September 2001 and 1 July 2018. After applying exclusion criteria for individuals missing a spirometry in the initiation interval or having inhaled corticosteroid/long-acting beta-agonist (ICS/LABA) initiation before first monitoring date, the analytic population included 9247 firefighters. Excluding individuals who initiated ICS/LABA for ≤2 years, a study population of 8530 firefighters was established to perform the main analyses.

The source population included 10168 World Trade Center (WTC)-exposed male firefighters who were actively employed by FDNY on 11 September 2001, consented to research, and had at least one routine medical monitoring exam between 11 September 2001 and 1 July 2018 (Figure 1). Since the FDNY WTC-exposed firefighter cohort was less than 1% female, women were not included from the source population into our study. 

Data on demographics, height, weight, smoking status, WTC exposure level (defined by initial arrival time to work at the WTC site), spirometry measurements, and respiratory symptoms were retrieved from the FDNY employee database (race, sex, and age) or were assessed during routine medical monitoring exams. Spirometry measurements included Forced Expiratory Volume in 1 s (FEV_1_) and FEV_1_/forced vital capacity ratio. Respiratory symptoms included self-reported wheeze, dyspnea, and provocability. Provocability was evaluated by asking about symptoms of cough, wheeze, dyspnea, or chest tightness when exposed to smoke, fumes, odors, dust, allergens, temperature or humidity extremes, or physical activity. When individuals reported at least one respiratory symptom of provocability on the medical monitoring questionnaire, they were considered as having provocability. 

### 2.2. ICS/LABA Treatment

Medication data were obtained from the FDNY electronic medical record (EMR) and the FDNY WTC health program claims database. The date of treatment initiation was defined as the earliest of either the first note in the EMR or the first prescription billed. Treatment duration was defined as the interval between first and most recent prescription fill dates of ICS/LABA. Individuals were considered as having been treated with ICS/LABA therapy when the duration was at least 2 years (*n* = 2162). 

### 2.3. Dataset Structure and Exclusions

Analytic datasets were organized into 2-year time intervals, starting 11 September 2001 and ending 1 July 2018. When multiple visits occurred in the same 2-year time interval, the first visit with available data was used. Individuals missing a spirometry measurement in their ICS/LABA initiation interval (*n* = 813) were excluded from the analyses. As shown in Figure 1, an additional 108 individuals who had an ICS/LABA initiation date before their first monitoring exam date were also excluded. After applying those exclusion criteria, the analytic population included 9247 firefighters. Additionally, 717 individuals who initiated ICS/LABA for ≤2 years were excluded (Figure 1). Among the remaining study population of 8530 firefighters,1629 individuals initiated ICS/LABA for >2 years between 11 September 2001–1 July 2018. During the subsequent intervals, individuals missing spirometry data on a particular visit were excluded from that interval (total of 1496 visits). The follow-up time ended at either date of ICS/LABA initiation, participants’ last monitoring exam (if retired), or the end of the study period (1 July 2018), whichever came first. Missing respiratory symptom data were imputed by using symptom data from the prior interval.

### 2.4. Statistical Analyses

Descriptive statistics were reported as proportions for categorical variables and means (with SD) for continuous variables, all of which met normality assumptions. Differences between groups were evaluated by chi-squared and *t*-tests, respectively.

Multinomial logistic model assessed the associations of the number of respiratory symptoms of wheeze, dyspnea, or provocability with ICS/LABA treatment (>2y, ≤2y vs. no ICS/LABA) as the outcome. The four-level symptom variable compared those that had one, two, or three respiratory symptoms versus none of those three. The models were adjusted for age, WTC exposure, retirement status and FEV_1_. Multivariable logistic regression was used to determine covariates associated with initiation of ICS/LABA treatment versus no ICS/LABA treatment, for each 2-year interval. Individuals who had already been prescribed ICS/LABA treatment were excluded from the analyses of the later intervals. We performed receiver operating characteristic (ROC) analysis for the logistic models, calculating the area under the curve (AUC). The covariates were chosen based on theory and different models were evaluated using the AUC of the model. 

Cox proportional hazards regression was used to assess the validity of the probability of ICS/LABA initiation generated by the earlier logistic models to predict future ICS/LABA initiation. The upper 10th percentile of the probability from a 2-year interval was used as the exposure variable. The follow-up time started the beginning of the intervals tested (2007 and 2015, respectively) and ended at the earliest actual ICS/LABA initiation, last monitoring exam, or the end of the intervals tested (11 September 2011 or 1 July 2018), whichever came first. We tested the proportional hazard assumption and all models met the assumption.

In a sensitivity analysis, we used only complete data available for the logistic models, without imputing missing data for respiratory symptoms from the prior interval (*n* = 8466).

In a second sensitivity analysis we included individuals that had ICS/LABA treatment for less than 2 years (*n* = 717 after exclusions) in the ICS/LABA treatment group (total *n* = 9247).

Data analyses were performed using SAS 9.4 (SAS Institute Inc. Cary, NC). Figures were made using SAS and R v3.6.0.2 (R Core Team. R: A Language and Environment for Statistical Computing, 2019; https://www.R-project.org).

## 3. Results

During the study period, 6901 individuals never initiated ICS/LABA and 1629 received ICS/LABA treatment for more than 2 years. The median treatment duration of this treated group was 8.5 years (IQR 5.0–12.5). The proportion of individuals that was persistent in ICS/LABA use > 2 years out of all ICS/LABA initiators was 1629/2346 (69.4%).

**Figure 2:**
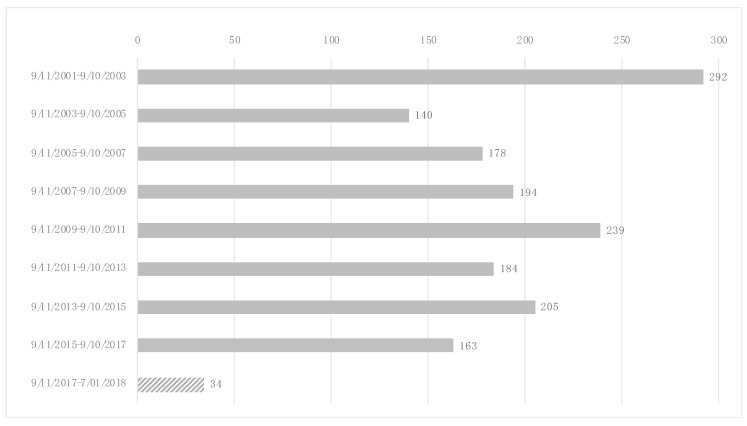
Distribution of individuals initiating ICS/LABA for more than 2 years (*n* = 1629) per time interval. The first interval, 11 September 2001–10 September 2003, with 292 individuals starting ICS/LABA treatment, had the highest number. In the other, full 2-year intervals the numbers of individuals initiating ranged from 139 to 239 per 2-year time interval. The last interval 11 September 2017–1 July 2018, which was not a full 2-year interval (striped filling pattern), contained 34 individuals with ICS/LABA initiation.

The number of individuals who initiated ICS/LABA in each 2-year time interval ranged between 34 and 292 (median 184 IQR 205–163) (Figure 2). The mean FEV_1_ around the time of ICS/LABA initiation was 87.7 ± 14.1% predicted and the FEV_1_/FVC ratio was 0.78 ± 0.07. Compared with nontreated individuals, a greater proportion of individuals who received ICS/LABA for more than 2 years arrived at the WTC site on the morning of 9/11 (19.5% vs. 15.0%) (Table 1). The 717 WTC exposed firefighters who were treated with ICS/LABA for ≤2 years had intermediate WTC exposure intensity, with 17.9% of treated individuals arriving on morning of 9/11.

### 3.1. Concurrent Symptoms and ICS/LABA Treatment Duration

We hypothesized that duration of treatment was associated with lung function and symptoms at the time of ICS/LABA initiation. To assess if patients with >2 years of ICS/LABA treatment were different from those with ≤2 years of ICS/LABA treatment, we performed multinomial logistic regression. As shown in Table 2, smaller expiratory lung volumes (FEV_1_) were more strongly associated with >2 years of ICS/LABA treatment than with ≤2 years of ICS/LABA treatment (OR 2.36 per L less, 95% CI 2.10–2.67 vs. 1.52, 95% CI 1.52–1.77). We also observed that concurrent symptoms were more strongly associated with >2 years of ICS/LABA treatment than with ≤2 years of ICS/LABA treatment. Compared with an asymptomatic patient, a patient with symptoms of wheeze, dyspnea, and provocability was 28-fold more likely (95% CI 23.2–35,8) to be treated with ICS/LABA for >2 years and 10-fold more likely (95% CI 7.6–13.3) to be treated with ICS/LABA for ≤2 years. 

### 3.2. Initiation of Prolonged ICS/LABA Treatment Over Time

**Figure 3:**
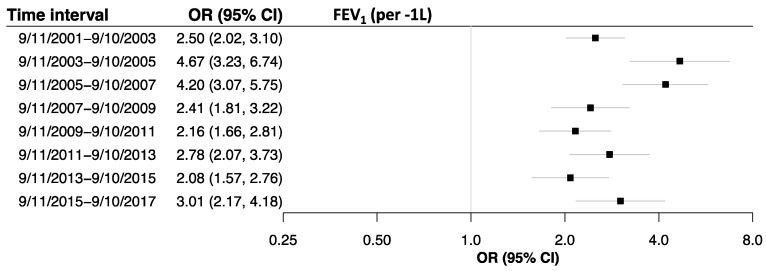

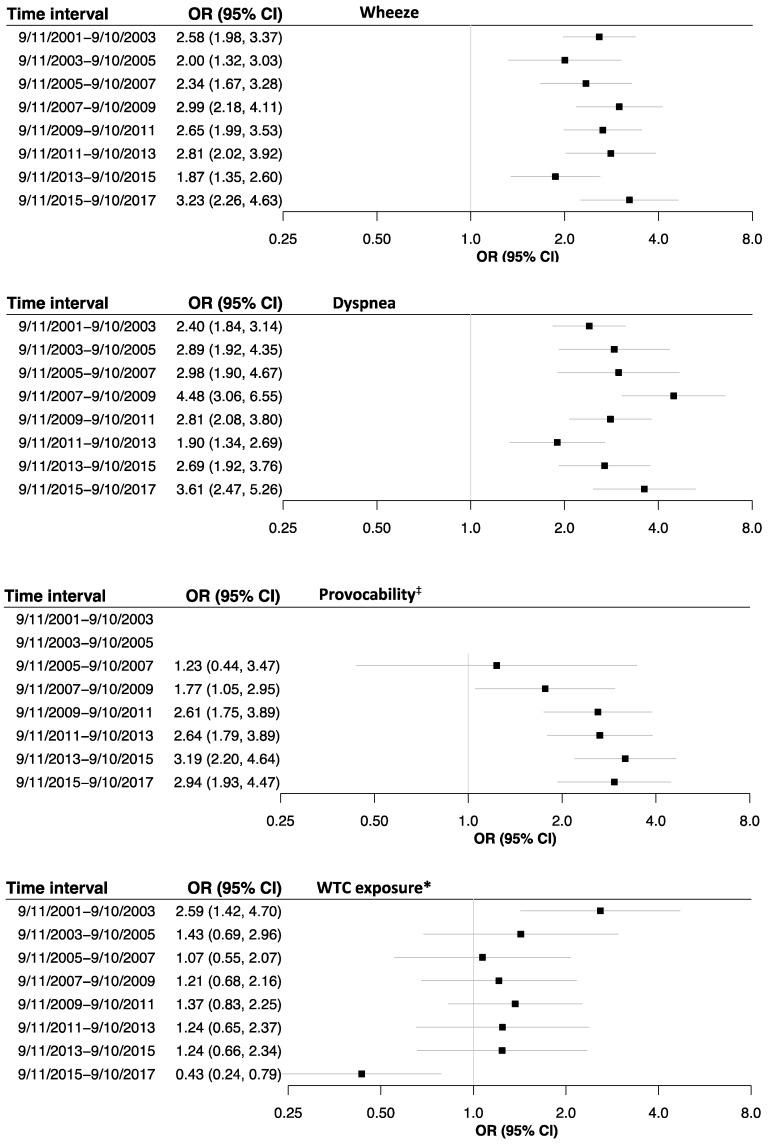
Forest plots showing variables associated with the initiation of inhaled corticosteroid/long-acting beta-agonist (ICS/LABA) treatment > 2 years between 11 September 2001 and 10 September 2017 (*n* = 1629) versus not initiating ICS/LABA treatment (*n* = 6901), created from multivariable logistic regression models examining the associations between medical monitoring exam covariates and initiation of prolonged ICS/LABA treatment (odds ratios (diamonds) and 95% confidence intervals (bars)). The models were adjusted for age and retirement status. Respiratory symptoms, such as wheeze, dyspnea, provocability, and lower FEV_1_, consistently predicted the early onset of lung injury defined by ICS/LABA treatment. High-intensity Word Trade Center exposure, based on arrival time, was strongly associated with ICS/LABA initiation soon after exposure. ‡ missing first two intervals because provocability data was not collected; * WTC exposure based on arrival at the WTC site: Morning of 11 September 2001 vs. 13 September 2001 or later.

After excluding the 717 individuals who were treated for ≤2 years and using patient characteristics measured on routine monitoring exams from each 2-year time interval over 16 years, we tested if factors associated with prolonged ICS/LABA treatment changed over time. Multivariable logistic regression demonstrated that wheeze, dyspnea, and lower FEV_1_ were persistent and independent correlates of ICS/LABA treatment (Figure 3). Symptoms of provocability were associated with ICS/LABA treatment after 11 September 2007. High-intensity WTC exposure, defined as arriving at the WTC site on the morning of 9/11, was associated with ICS/LABA treatment from 11 September 2001–10 September 2003 but not significantly thereafter. Age was not a meaningful predictor of treatment in any of the intervals (Table S1 in Supplementary Materials). 

We assessed ROC curves resulting from the models of each 2-year time interval. The AUC from the logistic models ranged from 0.77 (95% CI 0.73–0.80) to 0.85 (95% CI 0.81–0.88). 

We then tested if high probability of ICS/LABA treatment, based on the logistic regression model results, increased the hazard of ICS/LABA initiation in subsequent time intervals using Cox proportional hazards models (Table 3). The upper 10th percentile of risk for ICS/LABA initiation during the period 11 September 2005–10 September 2007 increased actual ICS/LABA treatment in the next interval by 3.32-fold (95% CI 2.58–4.26 *p* < 0.001) and in the 10 September 2015–1 July 2018 interval by 2.13-fold (95% CI 1.37–3.32 *p* ≤ 0.001).

### 3.3. Sensitivity Analyses

The results of the sensitivity analysis using only complete data for the logistic model (*n* = 8466) were similar to the primary analyses (Table S2 in Supplementary Materials).

A sensitivity analysis comparing individuals with ICS/LABA treatment of any duration (*n* = 2346) with never-treated individuals (*n* = 6901) demonstrated similar findings as the primary analyses (Table S3 in Supplementary Materials). 

## 4. Discussion

This retrospective cohort study identified factors predicting prolonged ICS/LABA treatment among WTC-exposed firefighters who were previously healthy when appointed to FDNY. Using longitudinal data from routine medical monitoring exams, we observed that wheeze, dyspnea, provocability, and lower FEV_1_ were independent risk factors for the early onset of lung injury defined by prolonged ICS/LABA treatment in patients with normal FEV_1_. There was a multiplicative increase in risk when two and three of the above symptoms co-occurred. We also demonstrated that factors present early in longitudinal follow-up increased the hazard for treatment initiation years later, suggesting that individuals with disease may have remained undiagnosed and untreated for significant periods of time. The diagnosis of occult disease as soon as possible is important, because in this cohort, delayed treatment was associated with poorer symptom control [10].

Wheeze and dyspnea, and lower FEV_1_ consistently predicted ICS/LABA initiation. Other factors, such as arrival at the WTC site on the morning of its collapse, provocability, and retirement status, changed over time. Using change-point models, we previously observed that the association of WTC exposure and obstructive airways disease have become attenuated over time [17]. Our observation that WTC exposure more strongly predicted ICS/LABA initiation shortly after is consistent with our prior studies.

The respiratory symptoms assessed in the study were derived from self-administered questionnaires and FEV_1_ measured with a handheld spirometer. These simple screening tests could be adapted to a telemedicine format. The importance of lung function and respiratory symptoms was recently studied with peak expiratory flow and asthma symptom scores in home telemonitoring to predict asthma exacerbations using machine learning algorithms [18]. As patient-reported outcomes, respiratory symptoms are gaining interest in clinical research [19]. Our observation that routine monitoring data predicted ICS/LABA treatment supports the utility of telemonitoring as a method to study pulmonary outcomes.

Patients who initiated ICS/LABA treatment but did not prolong it after 2 years were different than those who continued for more than 2 years, but these patients are interesting subgroup of individuals to further explore as well. Whereas reasons for their discontinuation are unclear, our results suggest this subgroup might also have increased risk for poor health outcomes based on their increased intensity of WTC exposure, wheeze, dyspnea, and lower lung volumes. It is also possible that this group consisted of both individuals who recovered well from their lung disease and therefore no longer needed treatment and individuals who would benefit of prolonged ICS/LABA treatment but failed to adhere to their chronic treatment. This latter group of individuals might especially benefit from increased monitoring of their symptoms and lung function over time to prevent further deterioration.

A potential weakness of our study is limited generalizability, because the FDNY cohort experienced a massive dust exposure and only included previously healthy males. We also acknowledge there may be unmeasured confounds, which is possible in all observational research. A strength of this investigation is comprehensive longitudinal data with little loss to follow-up, reducing the potential for selection bias. Drug use was objectively measured and the proportion of individuals who were persistent in ICS/LABA use > 2 years was 69%. This is slightly better than a study of real-world use of ICS/LABA in UK asthma patients, which found a proportion of patient persistence between 53% and 69% after only 1 year [20]. This slightly better adherence could be due to the increased monitoring and reimbursement of treatment within the WTC program.

Further research in occupational cohorts is needed to assess if routine respiratory symptom monitoring and hand-held spirometry can identify at-risk individuals who would benefit from subspecialty testing for obstructive airway disease.

## 5. Conclusions

In summary, we identified patient characteristics associated with ICS/LABA treatment. We observed that increased respiratory symptoms and lower FEV_1_ are persistent factors associated with treatment. 

Occupational cohorts that experience irritant exposures may require screening for obstructive airway disease with the goal to treat lung injury before development of abnormal lung function. 

## Figures and Tables

**Table 1:** Demographics comparing the full study population with the ICS/LABA-treated population.

Variable	Study Population *N* = 8530	ICS/LABA non-Treated *N* = 6901	ICS/LABA Treated >2 y *N* = 1629	ICS/LABA Treated ≤2 y *N* = 717
**Age on 9/11**	40.5 ± 7.5	40.4 ± 7.7	40.8 ± 6.8	39.8 ± 7.3
**Height (cm)**	177.1 ± 6.4	177.0 ± 6.4	177.2 ± 6.6	177.5 ± 6.6
Smoker, ever *	2195 (25.7)	1754 (25.4)	441 (27.1)	199 (27.8)
**Race**				
White	8008 (93.9)	6464 (93.7) ^‡^	1544 (94.8) ^‡^	677 (94.4)
Black	221 (2.6)	198 (2.9) ^‡^	23 (1.4) ^‡^	12 (1.7)
Other	301 (3.5)	239 (3.5) ^‡^	62 (3.8) ^‡^	28 (3.9)
**WTC exposure level**				
Morning of 9/11	1352 (15.9)	1034 (15.0) ^‡^	318 (19.5) ^‡^	128 (17.9)
Afternoon of 9/11 or 12 September 2001	6065 (71.1)	4908 (71.1) ^‡^	1157 (71.0) ^‡^	524 (73.1)
13-24 September 2001	1113 (13.1)	959 (13.9) ^‡^	154 (9.5) ^‡^	65 (9.1)
Baseline * FEV_1_ (L)	4.01 ± 0.68	4.06 ± 0.66 ^‡^	3.80 ± 0.68 ^‡^	3.97 ± 0.68
Baseline * FEV_1_ % predicted	97.3 ± 13.6	98.6 ± 13.1 ^‡^	91.8 ± 14.1 ^‡^	95.2 ± 14.2
Baseline * wheeze	1797 (21.1)	1210 (17.6) ^‡^	587 (36.0) ^‡^	216 (30.2)
Baseline * dyspnea	2454 (28.9)	1720 (25.0) ^‡^	734 (45.1) ^‡^	272 (38.0)

ICS/LABA = Inhaled steroids combined with long-acting beta-agonists; 9/11 = 11 September 2001; WTC = World Trade Center; * First post-9/11 exam; FEV_1_ = Forced Expiratory Volume in 1 s; ^‡^ *p* < 0.01 on *t*-tests and chi-squared.

**Table 2:** Multinomial logistic regression ^$^ for ICS/LABA treatment (*n* = 9018) *.

	ICS/LABA Treated > 2y *N* = 1570 *				ICS/LABA Treated ≤ 2y *N* = 690 *			
Variable	OR	95% CI		*p* Value	OR	95% CI		*p* Value
*FEV_1_ absolute ^#^, per -1 L*	2.36	2.10	2.67	<0.001	1.52	1.31	1.77	<0.001
Respiratory symptoms ^±^, 1 vs. 0	3.68	3.06	4.42	<0.001	2.57	2.07	3.20	<0.001
Respiratory symptoms ^±^, 2 vs. 0	10.04	8.37	12.05	<0.001	5.47	4.38	6.83	<0.001
Respiratory symptoms ^±^^,^ 3 vs. 0	28.80	23.19	35.76	<0.001	10.06	7.60	13.32	<0.001

^$^ Adjusted for age, WTC exposure, and retirement status. * *N* = 228 were excluded from the analysis due to missing covariates. (142 in ICS/LABA nontreated, 59 in >2y and 27 in ≤2y group). ICS/LABA nontreated (*N* = 6759) as the reference group; ^±^ Numbers of (concurrent) symptoms of wheeze, dyspnea, and/or provocability. ^#^ On medical monitoring exam closest to ICS/LABA initiation or most recent for non-ICS/LABA.

**Table 3:** Cox regression models for ICS/LABA treatment.

Probability * on ICS/LABA Treatment	Predicting	HR	95% CI		*p* Value
9/11/2005–9/10/2007	9/10/2007–9/11/2011	3.32	2.58	4.26	<0.001
9/11/2005–9/10/2007	9/10/2015–7/1/2018	2.13	1.37	3.32	<0.001

* Top 10th percentile of probability on ICS/LABA treatment.

## Supplementary Materials

The following are available online at https://www.mdpi.com/1660-4601/17/23/9056/s1, Table S1. Results from multivariable logistic regression models examining the associations between medical monitoring exam covariates and initiation of inhaled corticosteroid/long-acting beta-agonist (ICS/LABA) treatment >2 years between 09/11/2001 and 09/10/2017 (n=1,629) versus not initiating ICS/LABA treatment (n=6,901), Table S2. Results from first sensitivity analysis using only complete data, not imputing missing data for respiratory symptoms from the prior interval (n=8,466)., Table S3. Results from second sensitivity analysis including individuals that had inhaled corticosteroid/long-acting beta-agonist (ICS/LABA) treatment for less than 2 years (n=717) to the ICS/LABA treatment group.
