# Supplementary material for: Factors Predicting Treatment of World Trade Center-Related Lung Injury: A Longitudinal Cohort Study
Source: Int J Environ Res Public Health. 2020 Dec 4;17(23):9056. doi: 10.3390/ijerph17239056 (PMC7730939; doi:10.3390/ijerph17239056)
Supplement: Supplementary file 1 [file ijerph-17-09056-s001.docx]

**Supplementary Materials**

**Table S1.** Results from multivariable logistic regression models examining the associations between medical monitoring exam covariates and initiation of inhaled corticosteroid/long-acting beta-agonist (ICS/LABA) treatment >2 years between 09/11/2001 and 09/10/2017 (n=1,629) versus not initiating ICS/LABA treatment (n=6,901).

|  | **Odds ratios from logistic regression per 2-year interval^*^** | | | | | | | |
| --- | --- | --- | --- | --- | --- | --- | --- | --- |
| Variables | *9/11/2001-9/10/2003* | *9/11/2003-9/10/2005* | *9/11/2005-9/10/2007* | *9/11/2007-9/10/2009* | *9/11/2009-9/10/2011* | *9/11/2011-9/10/2013* | *9/11/2013-9/10/2015* | *9/11/2015-9/10/2017* |
| Total N used in the model^¶^ | *7045* | *4737* | *4632* | *6032* | *6510* | *6074* | *5789* | *5688* |
| N initiated ICS/LABA treatment^¶^ | *278* | *116* | *171* | *190* | *236* | *180* | *201* | *162* |
| FEV_1_ absolute, per -1L | 2.50 | 4.67 | 4.20 | 2.41 | 2.16 | 2.78 | 2.08 | 3.01 |
| Wheeze | 2.58 | 2.00 | 2.34 | 2.99 | 2.65 | 2.81 | 1.87 | 3.23 |
| Dyspnea | 2.40 | 2.89 | 2.98 | 4.48 | 2.81 | 1.90 | 2.69 | 3.61 |
| Provocability | na | na | (1.23) | 1.77 | 2.61 | 2.64 | 3.19 | 2.94 |
| WTC exposure, early vs late^#^ | 2.59 | (1.43) | (1.07) | (1.21) | (1.37) | (1.24) | (1.24) | 0.43 |
| WTC exposure, intermediate vs late^#^ | (2.21) | (0.92) | (1.23) | (0.98) | (1.00) | (1.39) | (1.64) | (0.53) |
| Age | (1.01) | (0.98) | (1.00) | (0.99) | (0.99) | (0.98) | (1.00) | (0.99) |
| Active status^±^ | $ | (0.41) | (0.87) | (1.10) | 1.54 | 1.91 | 3.11 | 4.74 |

9/11=9/11/2001; FEV_1_=Forced Expiratory Volume in 1 second; WTC=World Trade Center

Odds Ratio when p value < 0.05; (Odds Ratio) when p value > 0.05

*different intervals result from separate models;

¶individuals might be excluded from the analysis due to missing covariates;

# WTC exposure based on arrival at the WTC site: morning of 9/11 (early),

afternoon of 9/11 or 9/12/2001 (intermediate), vs 9/13 or later (late);

±not having retired in previous interval;

$estimate is 0 since all individuals were active on 9/11.

**Table S2.** Results from first sensitivity analysis using only complete data, not imputing missing data for respiratory symptoms from the prior interval (n=8,466). Multivariable logistic regression models examining the associations between medical monitoring exam covariates and initiation of inhaled corticosteroid/long-acting beta-agonist (ICS/LABA) treatment >2 years between 09/11/2001 and 09/10/2017 (n=1,629) versus not initiating ICS/LABA treatment (n=6,837).

|  | **Odds ratios from logistic regression per 2-year interval^*^** | | | | | | | |
| --- | --- | --- | --- | --- | --- | --- | --- | --- |
| Variables | *9/11/2001-9/10/2003* | *9/11/2003-9/10/2005* | *9/11/2005-9/10/2007* | *9/11/2007-9/10/2009* | *9/11/2009-9/10/2011* | *9/11/2011-9/10/2013* | *9/11/2013-9/10/2015* | *9/11/2015-9/10/2017* |
| Total N used in the model^¶^ | *7054* | *184* | *4630* | *6012* | *6469* | *6014* | *5729* | *5648* |
| N initiated ICS/LABA treatment^¶^ | *278* | *0*^†^ | *171* | *189* | *228* | *163* | *186* | *159* |
| FEV_1_ absolute, per -1L | 2.50 | - | 4.21 | 2.46 | 2.06 | 2.77 | 1.99 | 2.95 |
| Wheeze | 2.58 | - | 2.34 | 3.03 | 2.75 | 3.09 | 2.05 | 3.22 |
| Dyspnea | 2.40 | - | 2.98 | 4.61 | 3.00 | 2.00 | 2.70 | 3.60 |
| Provocability | na | - | (1.23) | 1.72 | 2.48 | 2.80 | 3.11 | 3.08 |
| WTC exposure, early vs late^#^ | 2.59 | - | (1.07) | (1.15) | (1.39) | (1.27) | (1.27) | (0.43) |
| WTC exposure, intermediate vs late^#^ | (2.21) | - | (1.23) | (0.96) | (1.01) | (1.47) | 1.61 | (0.51) |
| Age | (1.01) | - | (1.00) | (0.99) | (0.99) | (0.98) | (1.00) | (0.99) |
| Active status^±^ | $ | - | (0.87) | (1.07) | 1.51 | 1.72 | 3.06 | 4.65 |

9/11=9/11/2001; FEV_1_=Forced Expiratory Volume in 1 second; WTC=World Trade Center

Odds Ratio when p value < 0.05; (Odds Ratio) when p value > 0.05

*different intervals result from separate models;

¶individuals might be excluded from the analysis due to missing covariates;

†no individuals who initiated ICS/LABA having complete data, thus no model possible;

# WTC exposure based on arrival at the WTC site: morning of 9/11 (early),

afternoon of 9/11 or 9/12/2001 (intermediate), vs 9/13 or later (late);

±not having retired in previous interval;

$estimate is 0 since all individuals were active on 9/11.

**Table S3.** Results from second sensitivity analysis including individuals that had inhaled corticosteroid/long-acting beta-agonist (ICS/LABA) treatment for less than 2 years (n=717) to the ICS/LABA treatment group. Multivariable logistic regression models examining the associations between medical monitoring exam covariates and initiation of ICS/LABA treatment (n=2346) between 09/11/2001 and 09/10/2017 versus not initiating ICS/LABA treatment (n=6,901).

|  | **Odds ratios from logistic regression per 2-year interval^*^** | | | | | | | |
| --- | --- | --- | --- | --- | --- | --- | --- | --- |
| Variables | *9/11/2001-9/10/2003* | *9/11/2003-9/10/2005* | *9/11/2005-9/10/2007* | *9/11/2007-9/10/2009* | *9/11/2009-9/10/2011* | *9/11/2011-9/10/2013* | *9/11/2013-9/10/2015* | *9/11/2015-9/10/2017* |
| Total N used in the model^¶^ | *7672* | *5111* | *4947* | *6397* | *6865* | *6353* | *6023* | *5867* |
| N initiated ICS/LABA treatment^¶^ | *436* | *168* | *222* | *243* | *309* | *234* | *257* | *233* |
| FEV_1_ absolute, per -1L | 2.15 | 2.89 | 3.20 | 2.76 | 1.80 | 2.20 | 1.78 | 2.46 |
| Wheeze | 2.27 | 2.17 | 2.18 | 2.38 | 2.58 | 3.04 | 1.87 | 2.82 |
| Dyspnea | 2.45 | 2.21 | 2.37 | 3.58 | 2.93 | 1.88 | 2.62 | 3.18 |
| Provocability | na | na | (1.18) | 1.87 | 1.98 | 2.14 | 2.49 | 2.41 |
| WTC exposure, early vs late^#^ | 1.84 | (1.16) | (1.47) | (1.26) | (1.42) | (1.16) | (1.46) | (0.58) |
| WTC exposure, intermediate vs late^#^ | (1.61) | (0.93) | (1.33) | (1.13) | (1.09) | (1.39) | (1.65) | (0.67) |
| Age | 1.02 | (1.01) | (1.00) | (0.98) | (0.99) | (0.98) | (1.01) | 0.97 |
| Active status^±^ | $ | (0.32) | (0.96) | (1.01) | 1.37 | 1.79 | 2.98 | 3.81 |

9/11=9/11/2001; FEV_1_=Forced Expiratory Volume in 1 second; WTC=World Trade Center

Odds Ratio when p value < 0.05; (Odds Ratio) when p value > 0.05

*different intervals result from separate models;

¶individuals might be excluded from the analysis due to missing covariates;

# WTC exposure based on arrival at the WTC site: morning of 9/11 (early),

afternoon of 9/11 or 9/12/2001 (intermediate), vs 9/13 or later (late);

±not having retired in previous interval;

$estimate is 0 since all individuals were active on 9/11.
